# Adsorption of Uranyl Ions at the Nano-hydroxyapatite and Its Modification

**DOI:** 10.1186/s11671-017-2042-8

**Published:** 2017-04-18

**Authors:** Ewa Skwarek, Agnieszka Gładysz–Płaska, Yuliia Bolbukh

**Affiliations:** 1 Department of Radiochemistry and Colloid Chemistry, Faculty of Chemistry, Maria Curie Skłodowska University, M. Curie Skłodowska Sq. 3, 20-031 Lublin, Poland; 2 Department of Inorganic Chemistry, Faculty of Chemistry, Maria Curie Skłodowska University, M. Curie Skłodowska Sq. 2, 20-031 Lublin, Poland; 3 Nanomaterials Department, Chuiko Institute of Surface Chemistry of National Academy of Sciences of Ukraine, 17 General Naumov Str., Kyiv, 03164 Ukraine

**Keywords:** Adsorption U(VI), Nano-hydroxyapatite, Electrical double layer, 68.43.Mn, 82.70.Dd, 87.68.+z

## Abstract

Nano-hydroxyapatite and its modification, hydroxyapatite with the excess of phosphorus (P-HAP) and hydroxyapatite with the carbon ions built into the structure (C-HAP), were prepared by the wet method. They were studied by means of XRD, accelerated surface area and porosimetry (ASAP), and SEM. The size of crystallites computed using the Scherrer method was nano-hydroxyapatite (HAP) = 20 nm; P-HAP—impossible to determine; C-HAP = 22 nm; nano-HAP/U(VI) = 13.7 nm; P-HAP/U(VI)—impossible to determine, C-HAP/U(VI) = 11 nm. There were determined basic parameters characterizing the double electrical layer at the nano-HAP/electrolyte and P-HAP/electrolyte, C-HAP/electrolyte inter faces: density of the surface charge and zeta potential. The adsorption properties of nano-HAP sorbent in relation to U(VI) ions were studied by the batch technique. The adsorption processes were rapid in the first 60 min and reached the equilibrium within approximately 120 min (for P-HAP) and 300 min (for C-HAP and nano-HAP). The adsorption process fitted well with the pseudo-second-order kinetics. The Freundlich, Langmuir–Freundlich, and Dubinin–Radushkevich models of isotherms were examined for their ability to the equilibrium sorption data. The maximum adsorption capabilities (*q*_*m*_) were 7.75 g/g for P-HAP, 1.77 g/g for C-HAP, and 0.8 g/g for HAP at 293 K.

## Background

Apatite minerals occurring in the natural environment, e.g., rocks and coral sea, are durable for hundred millions of years under various ecological conditions. They are also produced synthetically by means of precipitation reactions and high-temperature processes. They can also be obtained from animal bones due to thermal treatment and as a result of the action of hydrogen peroxide in order to remove organic fractions from bones. Chemically appetites are similar to components of bones and hard tissues of mammals classified as biomaterials [1, 2].

Much effort is devoted to development of biomaterials based on nano-hydroxyapatite which have properly selected adsorption and adhesion properties by changing their surface with organic molecules possessing suitable binding groups. Surface modification is aimed at improvement of material effectiveness in the body fluid environment in humans and animals.

Apatite plays a significant role in both isolation of dissolved metals and their transformation in soil into less soluble phases. The literature presents the papers on strong affinity of apatite for strontium and other metals [3–5]. Apatite is an ideal material for long-term binding of contaminates due to its high sorption capacity for the actinide series and heavy metals, poor solubility in water, high stability under reducing and oxidizing conditions, accessibility, and low preparation cost.

Uranium is a heavy metal naturally occurring at the oxidation degrees: +6, +5, +4, +3, +2 but most frequently at +6. It can be found as a contaminant in the natural environment due to emissions from the nuclear industry, coal and other fuels burning, and natural weathering of magmatic rocks and ores which can penetrate the underground water filtering from hundreds to thousands of microgram per liter (ppb) of dilute uranium. Uranium appears immediately in the blood circulation whereas uranyl compounds combine readily with proteins and nucleotides forming stable complexes due to their strong affinity for phosphate ions as well as carboxyl and hydroxyl groups. The skeleton of mammals can be a main site of uranium accumulation in their organisms [6]. Also studies focusing on the adsorption of other ionic substances than uranium are presented in a literature [7, 8].

Therefore, studies on nano-hydroxyapatite [Ca_10_(OH)_2_(PO_4_)_6_] as a potential adsorbent for disinfection of uranium-contaminated underground waters can be of significant importance for people. Various minerals were studied as reactive materials as regards their capability of removing this dangerous metal from contaminated soils and waters: zeolites, iron on the zero oxidation degree, active carbon, and slaked lime. The scale of U(VI) removal from aqueous solutions depends on their properties such as pH of solution, concentration of uranium U(VI) complexing ligands, sorption phases, which are a kind of surface groups, number of sorption sites, etc. Knowledge of uranium removal mechanism is of significant importance in estimation of apatite effectiveness in immobilization of the contamination [9–11].

The investigation proposed in this paper will include measurements of uranyl ion adsorption at the nano-hydroxyapatite/electrolyte solution interface by means of spectroscopy method. The other aim of the paper, closely connected with the adsorption process, is the determination of the parameters characterizing the structure of double electrical layer formed at the nano-hydroxyapatite and its modification/electrolyte interface in the presence of uranyl ions.

## Methods

The studied adsorbents were synthesized by wet method. The studied samples were designated as follows: pure nano-hydroxyapatite—nano-HAP, hydroxyapatite with the excess of phosphorus—P-HAP, and hydroxyapatite with the carbon ions built into the structure—C-HAP.

The studies of carbonate apatite obtained by the wet method were carried out. In order to prepare reaction substrates, there were made 1 M solutions of the following reagents: Ca(OH)_2_ calcium hydroxide, 95% purity of the Aldrich firm, H_3_PO_4_—ortophosphoric acid (V), 85% purity of the POCh firm, and CaCO_3_ calcium carbonate prepared in the Department of Radiochemistry and Colloid Chemistry. Hydroxyapatite powder was obtained by titration of 180 cm^3^ of hydroxide suspension with phosphoric acid (V) solution pH = 9.2 were obtained. The procedure was repeated three times: on the average about 92 cm^3^ of H_3_PO_4_ was used. The analogous way was used to obtain HAP with incorporated carbonate groups. In this case, acid was used for titration of 200 cm^3^ of equimolar Ca(OH)_2_ and CaCO_3_ mixture (calcium carbonate was used in order to facilitate keeping of pH value about 9 with hydroxyapatite precipitation which is the most effective. The obtained sediments were washed many times with redistilled water and centrifuged till the constant value of conductivity of the solution from over precipitates was obtained and then dried at 80 °C for 24 h.

In case of phosphorus-enriched powders, appropriate solutions have been prepared: 0.12 M K_2_HPO_4_ (produced by POCh Gliwice) or 0.2 M (CH_3_COO)_2_Ca (produced by Riedel-de Haen). Both solutions has been dropped to 0.2 dm^3^ of redistilled water in reaction flask. The flask has been immersed in water batch heated to 100 °C. Salt solutions have been dropped simultaneously for 30 min., then the reaction mixture has been boiled for 1 h. All this time, vigorous stirring and stable temperature have been maintained. The obtained sediments were washed many times with redistilled water and centrifuged till the constant value of conductivity of the solution from over precipitates was obtained and then dried at 80 °C for 24 h.

Surface properties of nano-hydroxyapatite and its modification were studied by means of X-ray diffraction (XRD), adsorption-desorption of nitrogen (ASAP 2405 type Accelerated Surface Area and Porosimetry by the Micromeritics Instruments, Co firm) were investigated. The XRD radiation diffraction was studied by means of a diffractometer equipped with a rtg generator of the ISO- DEBYFLEX 303-60 kV type produced by the Seifert Analytical X-ray firm and a cooling system KMW 3000C produced by the Oxford Diffraction firm. The measurement data were collected, analyzed, and processed using the XRAYAN program. The morphology of composites was evaluated by scanning electron microscopy (SEM) using Quanta 3D FEG (FEI Co.).

Physicochemical properties chracteristic of electrical double layer (EDL) of nano-hydroxyapatite [Ca_10_(OH)_2_(PO_4_)_6_] surface were studied by means of potentiometric titration and electrophoretic measurements. Surface charge measurements were performed simultaneously in the suspension of the same solid content, to keep the identical conditions of the experiments in a thermostated Teflon vessel at 25 °C. To eliminate the influence of CO_2_, all potentiometric measurements were made under nitrogen atmosphere. The pH values were measured using a set of glass REF 451 and calomel pHG201-8 electrodes with the radiometer assembly. Surface charge density was calculated from the difference of the amounts of added acid or base to obtain the same pH value of suspension as for the background electrolyte. As a background electrolyte NaNO_3_ solution was used at the concentrations 0.1, 0.01, and 0.001 mol/dm^3^. The surface charge density and zeta potential were measured for the background electrolyte NaNO_3_ concentration (0.001 mol/dm^−3^) as a function of pH and concentration of the uranyl ions ranged from 0.01 to 0.000001 mol/dm^−3^. The zeta potential and size particles were determined by electrophoresis with Zetasizer 3000 by Malvern. The measurements were performed at 100 ppm solid concentration ultrasonication of the suspension.

Sorption of uranyl ions on nano-hydroxyapatite was studied by investigating sorption isotherms and the influence of pH.

### Adsorption Experiments

The adsorption experiments were performed using the batch method at 293, 313, and 333 K. The reaction mixtures (50 cm^3^) containing 10 mg of adsorbent and the solution of UO_2_(COOCH_3_)_2_·2H_2_O with the desired concentration of U(VI) ions were prepared. In the next step the mixtures were shaken for 2 or 24 h and filtered. The initial and equilibrium concentrations of U(VI) were determined spectrophotometrically using the Arsenazo III method [12]. The amount of U(VI) adsorbed on the sorbent was calculated from the difference between the initial and equilibrium concentrations from the equation:

1 $$ {c}_s=\left({c}_{\mathrm{in}}\hbox{-} {c}_{\mathrm{eq}}\right)\times \frac{V}{m}, $$

where *c*_*s*_ *, c*_*0*_ *,* and *c*_*eq*_ denote the concentrations of U(VI) in the sorbent phase as well as in the initial and equilibrium solutions. The symbols *V* and *m* relate to the volume of solution (dm^3^) and to the adsorbent mass (g).

The percentage adsorption of uranium from aqueous solution was computed as follows:

2 $$ A\left(\%\right)=\frac{100\%\times {c}_{in}-{c}_{eq}}{c_{in}}, $$

where *c*_*in*_ and *c*_*eq*_ are the initial and equilibrium uranium concentrations, respectively.

Kinetic experiments and pH influence were carried out at the initial uranium concentration of 0.5 mmol/dm^3^. The effect of pH was determined by studying the adsorption of uranium ions over a pH range 2–12. The pH was adjusted by the addition of HNO_3_ or NaOH solution. The pH values of the equilibrium solutions were controlled using a combined glass electrode (Sigma Chemical Co.) connected to the pH meter (CX-731 type, Elmetron Co.). All the experimental data were the averages of triplicate determinations. The relative errors of the data were about 2–3%.

## Results and Discussion

**Fig. 1:**
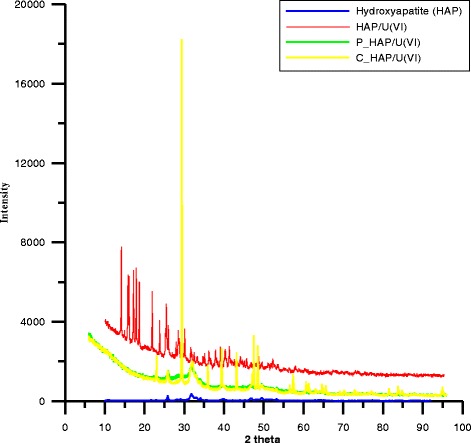
X-ray diffraction pattern for the nano-hydroxyapatite sample and its modification with adsorbed uranyl nitrate

The XRD data confirms phase purity of the studied samples (Fig. 1.) Peak characteristic of crystal form of nano-hydroxyapatite, i.e., peaks and their intensities occurring at the angles 2θ 25.9–35%; 31.75–100%; 32.96–55%; 39.84–20%; 46.7–40%; 49.5–30%, can be observed. This is in agreement with the phase analysis made from the ASTM data. As follows from the XRD analysis after U(VI) adsorption on the nano-hydroxyapatite surface, there is formed a new compound calcium uranyl phosphate hydrate Ca(UO_2_)_2_(PO_4_)_2_ (H_2_O)_11_ on the surface in the amount of 68.9% but 31% is nano-hydroxyapatite(red curve). The formed sediment is more stable in the acidic and inert environment, correlates well with the results of adsorption described later, and confirms possibility of using nano-hydroxyapatite for removal of 6-valued uranium from aqueous solutions. The sample HAP-P is low crystalline and amorphous also after U(VI) ion adsorption has an amorphous form. In another C-HAP/U(VI) sample (yellow curve), a new compound 51% CaCO_3_ and 47% nano-HAP were formed on the surface due to adsorption. The size of crystallites computed using the Scherrer method was nano-HAP = 20 nm; P-HAP—impossible to determine; C-HAP = 22 nm; nano-HAP/U(VI) = 13.7 nm; P-HAP/U(VI)—impossible to determine, C-HAP/U(VI) = 11 nm.

The parameters of porous structure of nano-hydroxyapatites were determined using the standard method which is low-temperature nitrogen adsorption/desorption. The measurements were made after samples degassing and reduced pressure, and the results are shown in Tables 1-3.

**Table 1:** Chosen structural parameters for nano-HAP and nano-HAP/U(VI)

Parameter	Nano-HAP	Nano-HAP/U(VI)
BET surface area [m^2^ g^−1^]	105	25
Langmuir surface area [m^2^ g^−1^]	134	37
BJH cumulative adsorption surface area of pores between 1.7 and 300 nm diameter [cm^3^ g^−1^]	0.54	0.13
BJH cumulative desorption surface area of pores between 1.7 and 300 nm diameter [cm^3^ g^−1^]	0.53	0.13
Average pore diameter (4V/A by BET) [nm]	17.72	20.36
BJH adsorption on average pore diameter (4V/A) [nm]	18.08	20.75
BJH desorption on average pore diameter (4V/A) [nm]	17.46	13.51

As follows from the results (Table 1), U(VI) adsorbed on the surface causes multiple decrease in the specific surface area and decrease in the average radius of the pores which may be due to formation of calcium uranyl phosphate hydrate layer on the nano-hydroxyapatite layer as confirmed by the XRD studies. The specific surface area decreases also after U(VI) adsorption for the system P-HAP, stopping the P-HAP pores (Table 2). The specific surface area does not change only for the system C-HAP and C-HAP/U(VI) and the volume of pores increases (Table 3) which may be due to formation of CaCO_3_ that results in Ca ions escape from the surface of hydroxyapatites causing at the same time a pore volume increase. The amount of Ca ions is the largest at the surface in the nano-hydroxyapatite structure compared to other elements building it.

**Table 2:** Chosen structural parameters for P-HAP and P-HAP/U(VI)

Parameter	P-HAP	P-HAP/U(VI)
BET surface area [m^2^ g^−1^]	85	61
Langmuir surface area [m^2^ g^−1^]	125	89
BJH cumulative adsorption surface area of pores between 1.7 and 300 nm diameter [cm^3^ g^−1^]	0.43	0.27
BJH cumulative desorption surface area of pores between 1.7 and 300 nm diameter [cm^3^ g^−1^]	0.43	0.28
Average pore diameter (4V/A by BET) [nm]	20.26	18.28
BJH adsorption on average pore diameter (4V/A) [nm]	20.01	19.39
BJH desorption on average pore diameter (4V/A) [nm]	14.03	15.24

**Table 3:** Chosen structural parameters for C-HAP and C-HAP/U(VI)

Parameter	C-HAP	C-HAP/U(VI)
BET surface area [m^2^ g^−1^]	44	44
Langmuir surface area [m^2^ g^−1^]	66	64
BJH cumulative adsorption surface area of pores between 1.7 and 300 nm diameter [cm^3^ g^−1^]	0.27	0.32
BJH cumulative desorption surface area of pores between 1.7 and 300 nm diameter [cm^3^ g^−1^]	0.27	0.33
Average pore diameter (4V/A by BET) [nm]	24.40	29.8
BJH adsorption on average pore diameter (4V/A) [nm]	24.87	32.17
BJH desorption on average pore diameter (4V/A) [nm]	23.53	29.20

**Fig. 2:**
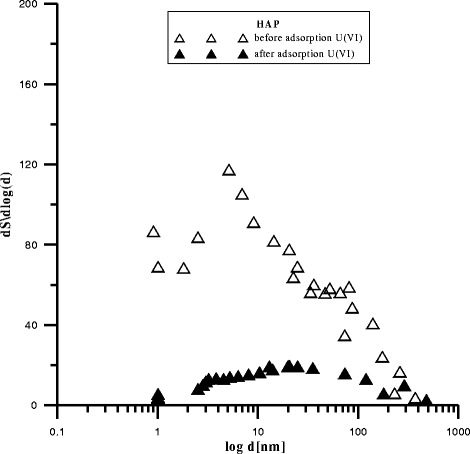
Pores in HAP before and after adsorption U(VI)

**Fig. 3:**
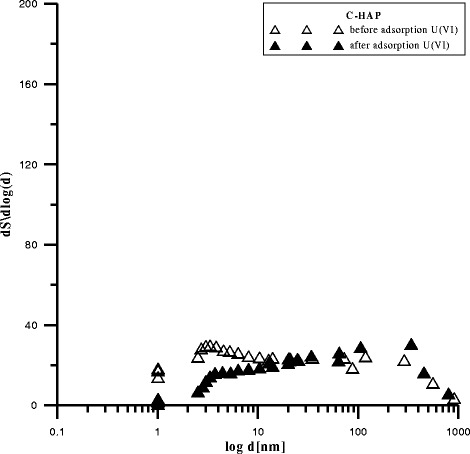
Pores in C-HAP before and after adsorption U(VI)

**Fig. 4:**
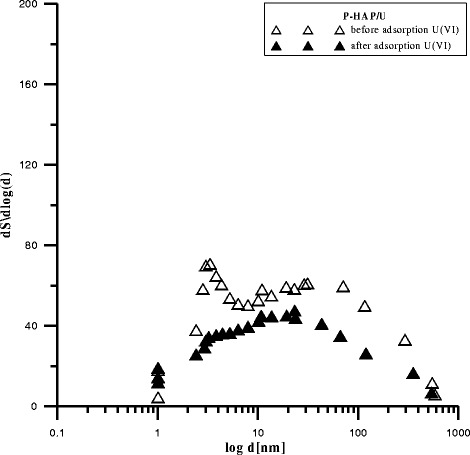
Pores in P-HAP before and after adsorption U(VI)

The data in Figs. 2, 3, and 4 presenting the differential pore distribution indicate that HAP, C-HAP, and P-HAP contain micropores, mezopores, and macropores. After U(VI) adsorption, the number of mesopores and macropores increased and that of micropores decreased which is in agreement with the results presented in the table earlier.

**Fig. 5:**
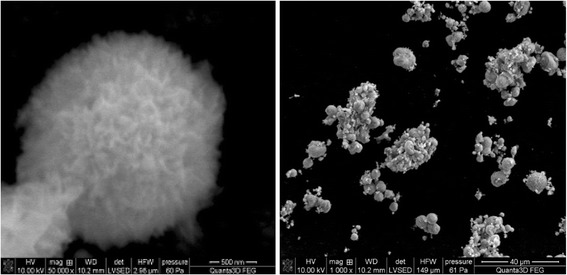
SEM images of the HAP

**Fig. 6:**
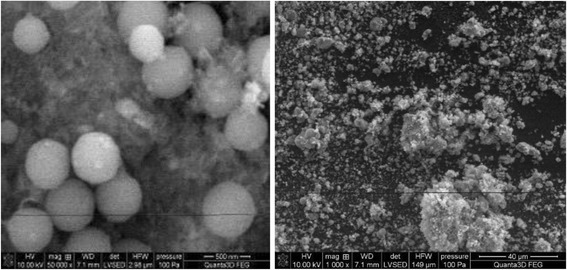
SEM images of the P-HAP.

**Fig. 7:**
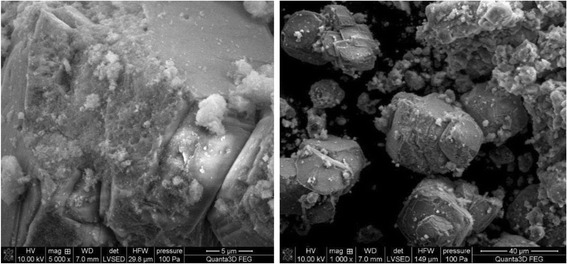
SEM images of the C-HAP

The data in Figs. 5, 6, and 7 presenting the SEM images indicate that materials HAP, C-HAP, and P-HAP have different morphology of the particles. Namely, an excess phosphorus leads to the formation in the volume of the powder spheres of 300–500 nm and a smooth surface. The composite with embedded carbon is formed as a monolith whose particle size depends on the degree of grinding mostly.

The average size of particles of studied compounds determined by Zetasizer 3000 was as follows: HAP = 30 nm, P-HAP = 45 nm, and C-HAP = 62 nm.

**Fig. 8:**
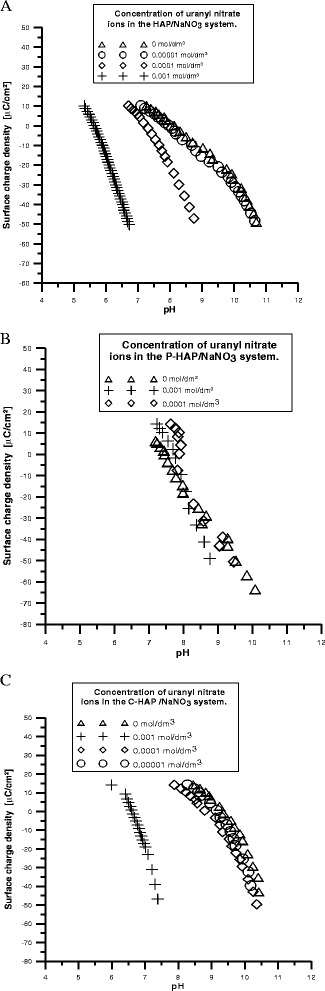
The surface charge density as a function of pH for the nano-hydroxyapatite (**a**), P-HAP (**b**) and C-HAP (**c**) dispersed in NaNO_3_ solution with different concentrations of U(VI)

Basic parameters of the electrical double layer were determined in order to study the behavior of nano-HAP, P-HAP, and C-HAP in the aqueous solution. The zero charge point pH_pzc_ value for the studied systems is pH_pzc_ = 6.5 for nano-HAP; pH_pzc_ = 7.5 for P-HAP, and pH_pzc_ = 8 for C-HAP. The increase in this parameter can be observed compared to the stoichiometric nano-HAP, which can be explained by incorporation of more acidic phosphate and carbon groups into the nano-hydroxyapatite structure probably due to their appearance instead of hydroxyl groups which contributes to the increase of U(VI) ion adsorption. Figure 8a, b, c presents the dependence of surface charge density on pH. The results indicate that surface charge density decreases with the increasing pH value for all studied concentration and have a negative value in almost whole pH range. The pH_pzc_ point changes with the increasing U(VI) concentration shifting towards acidic value as a result of U(VI) accumulation on the surface.

The pH_IEP_ value for the studied systems is pH_IEP_ <5 for nano-HAP; pH_IEP_ <4 for P-HAP, and pH_IEP_ <4 for C-HAP. The discrepancy between pH_pzc_ and pH_IEP_ for individual samples of the types, nano-HAP, P-HAP, and C-HAP, is caused by determination of surface charge density from the acidic-basic reaction of the surface groups of amphoteric hydroxyl ions and PO_4_^−3^ groups of acidic character. Additionally, the zeta potential depends on part of surface charge affected by no uniform absorption or desorption of calcium or phosphate ions.

**Fig. 9:**
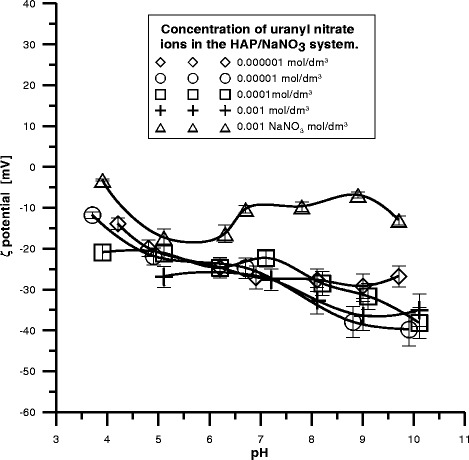
The zeta potential as a function of pH for the nano-hydroxyapatite (HAP) dispersed in NaNO_3_ solution with different concentrations of U(VI)

Dependence of zeta potential on pH for nano-hydroxyapatite was determined in NaNO_3_ of the concentration 0.001 mol/dm^3^ + 0.00001 mol/dm^3^; 0.0001 mol/dm^3^; 0.001 mol/dm^3^ U(VI) as shown in Fig. 9. As follows dependence of zeta potential on pH in the nano-hydroxyapatite NaNO_3_/U(VI) system with the increasing pH value, the zeta potential decreases and has negative values that take place in the range of 0 to −40 mV for the nano-hydroxyapatite/NaNO_3_/U(VI) system. Extrapolation of the zeta potential dependence in the pH function allows to suppose that the value of pH_IEP_ is <3. The zeta potential is dependent on this part of surface charge which causes that PO_4_^3−^ or Ca^2+^ ions adsorb or desorb at the nano-hydroxyapatite/solution interface. U(VI) ions affect the zeta potential value and compared to the initial electrolyte they decrease it in the whole pH range. However, for the concentrations from 0.001 to 0.000001 mol/dm^3^, they assume very similar values.

**Fig. 10:**
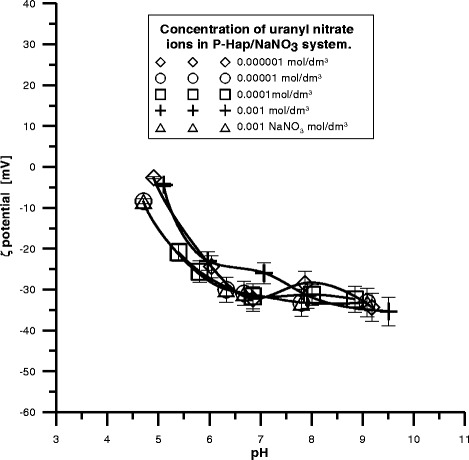
The zeta potential as a function of pH for P-HAP dispersed in NaNO_3_ solution with different concentrations of uranyl nitrate ions

**Fig. 11:**
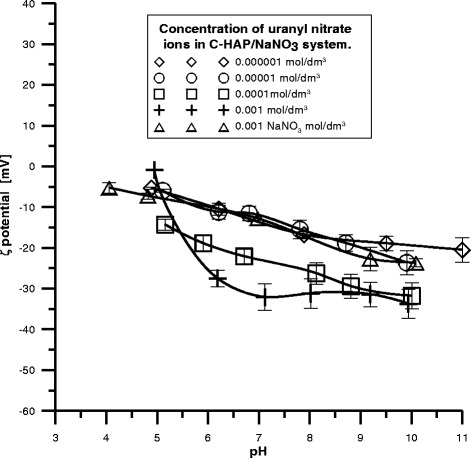
The zeta potential as a function of pH for C-HAP dispersed in NaNO_3_ solution with different concentrations of U(VI)

The diagrams of zeta potential dependence on pH for the P-HAP/U(VI) and C-HAP/U(VI) systems contain a definite amount of U(VI) are presented in Figs. 10 and 11. Comparing these figures one can see that the potential changes after the addition of U(VI) ions. At higher pH values evident decrease in the zeta potential with the increasing U(VI) ions concentration at constant pH is observed. The larges effect of concentration changes on the zeta potential can be seen in Fig. 11 for the C-HAP system which may be due to a larger difference in the atomic radius between uranium equal to 175 pm and carbon equal to 70 pm thus stronger repulsion of ions in the U(VI) solution and CO_3_ releasing from the surface.

### Adsorption Kinetic Models

**Fig. 12:**
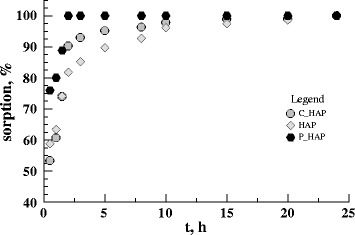
The kinetics of U(VI) adsorption on the  HAP sorbents (293 K; *c*_in_U(VI) = 0.5 mmol × dm^−3^; pH = 6)

**Fig. 13:**
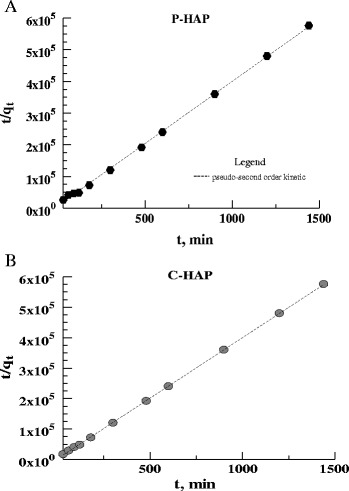
The kenetics of U(VI) absorption on the P-HAP (**a**) and C-HAP (**b**) sorbents

The equilibrium of uranium adsorption on the sorbents is established after 120 min from the beginning of the process (Figs. 12 and 13). The adsorption of uranium ions quickly reaches 98% and then achieves plateau of 99% after 24 h. Two kinetic models have been used to characterize sorption of uranium ions on the sorbents:

the pseudo-first-order kinetic equation

3 $$ \log \left({q}_{\mathrm{e}}-{q}_{\mathrm{t}}\right)= \log \kern0.5em {q}_{\mathrm{e}}-\frac{k_1}{2.303}\times t $$

and the pseudo-second-order kinetics equation

4 $$ \frac{t}{q_t}=\frac{1}{k_2{q}_e^2}+\left(\frac{1}{q_e}\right)\times t, $$

The linear plots of *t*/*qt* vs. *t* were used to calculate the kinetic parameters. The constants contained in these equations represent the following: q_e_ and q_t_ are the amounts of uranium ions adsorbed at equilibrium in mg × g^−1^, and at time *t* in min, *k*1 and *k*2 are the pseudo-first-order rate constant (min^−1^) and the second-order rate constant of adsorption (g × mg^−1^ × min^−1^), respectively. The calculated values of these constants are listed in Table 4. The adsorption of uranium ions on the sorbents followed by pseudo-second-order kinetics, and this proves chemisorption as a major mechanism [13–15].

**Table 4:** Parameters of the kinetic models for the adsorption of the uranium ions on the HAP sorbent

Model	Parameter	C-HAP	P-HAP	HAP
Pseudo-first-order	*k*_1_ (min^−1^)	0.00022	0.004	0.00011
	*q*_e_calc (mol × g^−1^)	9.2 × 10^−7^	9.3 × 10^−7^	9.12 × 10^−7^
	*R* ^2^	0.5567	0.6577	0.7907
Pseudo-second-order	*k*_2_ (g × mo^−1^ × min^−1^)	45.55	22.13	11.52
	*q*_e_calc (mol × g^−1^)	0.0025	0.00251	0.00254
	*R* ^2^	0.9997	0.9998	0.9999

*R* ^2^ correlation

#### Adsorption Isotherms

**Fig. 14:**
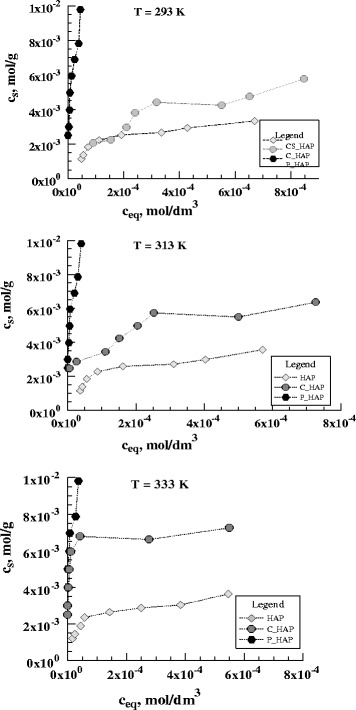
The isotherms of U(VI) adsorption on HAP sorbents (pH_in_ = 6; *T* = 293 K, 313 K, 333 K) *c*_*s*_ concentration of U(VI) in the sorbent phase (mol/g), *c*_*eq*_ equilibrium concentration of U(VI) in the aqueous phase (mol × dm^−3^)

Figure 14 shows the adsorption isotherms of uranyl ions at three temperatures (293, 313, and 333 K) for different forms of sorbent. The highest sorption and almost non-temperature dependent has been reported for P-HAP, which is probably because of formation of the uranyl ion species with phosphorus and/or precipitation of the uranyl phosphate.

The equilibrium data were analyzed using the Langmuir–Freundlich, Freundlich, and Dubinin–Radushkevich models of isotherm. The Freundlich equation can be applied to multilayer adsorption, with non-uniform distribution of adsorption heat and affinities over the heterogeneous surface. This model isotherm is widely applied in heterogeneous systems especially for organic compounds or highly interactive species on activated carbon and molecular sieves.

The Langmuir–Freundlich isotherm is expressed by the following equation:

5 $$ {c}_{\mathrm{s}}=\frac{a{\left({K}_{\mathrm{L}\hbox{-} \mathrm{F}}\times {c}_{\mathrm{eq}}\right)}^n}{\left[1+{\left({K}_{\mathrm{L}\hbox{-} \mathrm{F}}\times {c}_{\mathrm{eq}}\right)}^n\right]} $$

The Freundlich model can be represented by linear form, also used for analyzing the experimental sorption data

6 $$ \log {c}_{\mathrm{s}}= \log {K}_{\mathrm{F}}+\frac{1}{n}{c}_{\mathrm{eq}} $$

where *K*_F_ and *K*_L-F_ is the Freundlich or the Langmuir–Freundlich isotherm constant (dm^3^ × mol^−1^), respectively, *n* is the heterogeneity parameter of the surface, and *a* is the adsorption maximum (mol × g^−1^). The parameter, *n*, is a measure of adsorption intensity or surface heterogeneity, and it is becoming more heterogeneous as its value gets closer to zero. The Langmuir–Freundlich and Freundlich parameters and the corresponding correlation coefficients (*R* ^2^) are summarized in Tables 5, 6, and 7. The sorption capacity of each sorbents reached from the Langmuir–Freundlich model is 7.75 g × g^−1^ for P-HAP, 1.77 g × g^−1^ for C-HAP, and 0.8 g × g^−^1 for nano-HAP at 293 K.

**Table 5:** Parameters of the isotherm models for the adsorption of U(VI) ions on the P-HAP

Model	Parameter	293 K	313 K	333 K
Langmuir–Freundlich	*K*_L-F_ (dm^3^ × mol^−1^)	220.73	1748.3	1222.2
	*n*	0.422	0.392	0.281
	*A* (mol × g^−1^)	3.26 × 10^−2^	3.5 × 10^−2^	3.32 × 10^−2^
	*R* ^2^	0.9494	0.9489	0.9255
Dubinin–Radushkevich	*K*_D-R_ (mol^2^/kJ^2^)	2.96 × 10^−9^	2.08 × 10^−9^	1.21 × 10^−9^
	*Q*_m_ (mol × g^−1^)	1.12 × 10^−3^	2.75 × 10^−3^	1.19 × 10^−3^
	*E*_n_ (kJ × mol^−1^)	12.99	15.5	16.33
	*R* ^2^	0.9898	0.9655	0.9267
Freundlich	*K*_F_ (dm^3^ × mol^−1^)	4.35	2.34	0.815
	*n*	0.38	0.32	0.21
	*R* ^2^	0.9494	0.9498	0.9259

*R* ^2^ correlation

**Table 6:** Parameters of the isotherm models for the adsorption of U(VI) ions on the C-HAP

Model	Parameter	293 K	313 K	333 K
Langmuir–Freundlich	*K*_L-F_ (dm^3^ × mol^−1^)	3398.73	1092.3	4149.2
	*n*	0.904	0.332	1.13
	*A* (mol × g^−1^)	7.45 × 10^−3^	1.27 × 10^−2^	6.9 × 10^−3^
	*R* ^2^	0.9464	0.9425	0.9615
Dubinin–Radushkevich	*K*_D-R_ (mol^2^ × kJ^−2^)	4.73 × 10^−9^	1.53 × 10^−9^	6.2 × 10^−10^
	*Q*_m_ (mol × g^−1^)	1.66 × 10^−3^	2.57 × 10^−3^	2.3 × 10^−3^
	*E*_n_ (kJ × mol^−1^)	10.28	18.1	18.4
	*R* ^2^	0.9475	0.9345	0.8930
Freundlich	*K*_F_ (dm^3^ × mol^−1^)	0.11	0.0314	0.0136
	*n*	0.423	0.222	0.0821
	*R* ^2^	0.9411	0.9498	0.9213

*R* ^2^ correlation

**Table 7:** Parameters of the isotherm models for the adsorption of U(VI) ions on the HAP, (*R* ^2^ – correlation)

Model	Parameter	293 K	313 K	333 K
Langmuir–Freundlich	*K*_L-F_ (mol^2^ × kJ^−2^)	13524.54	15123.8	16724.8
	*n*	9.17	0.963	0.645
	*A* (mol × g^−1^)	3.37 × 10^−3^	3.6 × 10^−3^	4.21 × 10^−3^
	*R* ^2^	0.9808	0.9686	0.979
Dubinin–Radushkevich	*K*_D-R_ (mol^2^ × kJ^−2^)	3.53 × 10^−9^	2.87 × 10^−9^	2.02 × 10^−9^
	*Q*_m_ (mol × g^−1^)	2.95 × 10^−3^	2.82 × 10^−3^	1.78 × 10^−3^
	*E*_n_ (kJ × mol^−1^)	11.9	13.2	15.8
	*R* ^2^	0.9523	0.9518	0.9742
Freundlich	*K*_F_ (dm^3^ × mol^−1^)	0.0349	0.0354	0.025
	*n*	0.318	0.31	0.259
	*R* ^2^	0.9595	0.9582	0.975

The Dubinin–Radushkevich isotherm is an empirical model that is often used to express the adsorption mechanism with a Gaussian energy distribution onto a heterogeneous surface [16]. The model is represented by the following equation:

7 $$ {c}_{\mathrm{s}}=\kern0.5em {Q}_{\mathrm{m}}\times \kern0.5em  \exp \left(-{K}_{\mathrm{D}-\mathrm{R}}{\left( RT \ln \left(1+\frac{1}{c_{\mathrm{eq}}}\right)\right)}^2\right), $$

where *Q*_m_ is the model constant (mol × g^−1^), *K*_D-R_ is the Dubinin–Radushkevich isotherm constant (mol^2^ × kJ^-2^), *R* is the gas constant, and *T* stands for the temperature.

The free energy of adsorption, *E*_n_, can be calculated by the relationship:

8 $$ {E}_{\mathrm{n}}=\frac{1}{{\left(2{K}_{\mathrm{D}\hbox{-} \mathrm{R}}\right)}^{0.5}} $$

The energy values (Tables 5, 6, and 7) calculated by the Dubinin–Radushkevich equation are in the range characteristic of the chemisorption mechanism 8 < *E*_n_ < 16 kJ × mol^−1^ [17].

## Conclusions

The nano-hydroxyapatite (HAP) and its modification P-HAP, C-HAP samples obtained using the wet method, with pH control. It was found the specific surface areas for the samples decrease due to the presence of U(VI) which stops the nano-hydroxyapatite pores. Substitution of carbonate ions (C-HAP) and phosphate (P-HAP) affected significantly the structure and properties of nano-hydroxyapatite and U(VI) adsorption. The quantities characterizing the double electrical layer were measured for the studied systems; there were obtained the following values: pH_pzc_ = 6.5 for HAP, pH_pzc_ = 7.5 for P-HAP, and pH_pzc_ = 8 for C-HAP. The value pH_IEP_ for the studied systems is as follows: pH_IEP_ <5 for HAP; pH_IEP_ <4 for P-HAP, and pH_IEP_ <4 for C-HAP. The presence of U(VI) affects the size of surface charge density and zeta potential at the nano-hydroxyapatite and its modification/electrolyte solution interface. P-HAP proved to be the most effective adsorbents for the removal of uranium ions. Kinetic evaluation of the equilibrium data showed that the adsorption of U(VI) on the sorbent follows well the pseudo-second-order kinetic model. The adsorption energy evaluated on the basis of the Dubinin–Radushkevich equation for the sorbent is in the range 11–19 kJ × mol^−1^ which indicates that the process of adsorption of uranium ions is chemical in nature. Particularly, P-HAP seems to be a promising material for disinfection of water reservoirs from uranium because of its great affinity for uranium and formation of uranium phosphates of poor solubility.

## Abbreviations

ASAP: Accelerated surface area and porosimetry
C-HAP: Hydroxyapatite with the carbon ions built into the structure
EDL: Electrical double layer
HAP: Hydroxyapatite
IEP: Isoelectric point
P-HAP: Hydroxyapatite with the excess of phosphorus
PZC: Point of zero charge
SEM: Scanning electron microscopy
XRD: X-ray diffraction
